# Exploring Stakeholder Requirements to Enable the Research and Development of Artificial Intelligence Algorithms in a Hospital-Based Generic Infrastructure: Protocol for a Multistep Mixed Methods Study

**DOI:** 10.2196/42208

**Published:** 2022-12-16

**Authors:** Lina Weinert, Maximilian Klass, Gerd Schneider, Oliver Heinze

**Affiliations:** 1 Institute of Medical Informatics Heidelberg University Hospital Heidelberg Germany; 2 Section for Translational Health Economics Department for Conservative Dentistry Heidelberg University Hospital Heidelberg Germany

**Keywords:** artificial intelligence, requirements analysis, mixed methods, innovation, qualitative research, health care, artificial intelligence technology, diagnostic, health data, artificial intelligence infrastructure, technology development

## Abstract

**Background:**

In recent years, research and developments in advancing artificial intelligence (AI) in health care and medicine have increased. High expectations surround the use of AI technologies, such as improvements for diagnosis and increases in the quality of care with reductions in health care costs. The successful development and testing of new AI algorithms require large amounts of high-quality data. Academic hospitals could provide the data needed for AI development, but granting legal, controlled, and regulated access to these data for developers and researchers is difficult. Therefore, the German Federal Ministry of Health supports the Protected Artificial Intelligence Innovation Environment for Patient-Oriented Digital Health Solutions for Developing, Testing, and Evidence-Based Evaluation of Clinical Value (pAItient) project, aiming to install the AI Innovation Environment at the Heidelberg University Hospital in Germany. The AI Innovation Environment was designed as a proof-of-concept extension of the already existing Medical Data Integration Center. It will establish a process to support every step of developing and testing AI-based technologies.

**Objective:**

The first part of the pAItient project, as presented in this research protocol, aims to explore stakeholders’ requirements for developing AI in partnership with an academic hospital and granting AI experts access to anonymized personal health data.

**Methods:**

We planned a multistep mixed methods approach. In the first step, researchers and employees from stakeholder organizations were invited to participate in semistructured interviews. In the following step, questionnaires were developed based on the participants’ answers and distributed among the stakeholders’ organizations to quantify qualitative findings and discover important aspects that were not mentioned by the interviewees. The questionnaires will be analyzed descriptively. In addition, patients and physicians were interviewed as well. No survey questionnaires were developed for this second group of participants. The study was approved by the Ethics Committee of the Heidelberg University Hospital (approval number: S-241/2021).

**Results:**

Data collection concluded in summer 2022. Data analysis is planned to start in fall 2022. We plan to publish the results in winter 2022 to 2023.

**Conclusions:**

The results of our study will help in shaping the AI Innovation Environment at our academic hospital according to stakeholder requirements. With this approach, in turn, we aim to shape an AI environment that is effective and is deemed acceptable by all parties.

**International Registered Report Identifier (IRRID):**

DERR1-10.2196/42208

## Introduction

### Background

The advancement and development of artificial intelligence (AI) in health care hold several promises, such as the heightened quality of diagnosis and treatment [1-4], improvements for clinical workflow processes [5], and a reduction of costs [3,6]. These hopes also apply to health systems’ reactions to global health emergencies, such as the COVID-19 pandemic or future challenges [7]. For the purpose of this research, we used the definition by He et al [8], who define AI as a “branch of applied computer science wherein computer algorithms are trained to perform tasks typically associated with human intelligence” [8].

Although AI development and research are conducted in many countries nowadays [5], a report from the Joint Research Centre of the European Commission [9], as well as original research, shows that AI development and implementation in the European Union and in Germany are still in their early stages [10,11]. In part, this could be explained by the low availability of the data needed for the development of AI tools [12,13] and by regulatory and legal uncertainties [10]. In its efforts to support the further development of AI tools within the country, the German government deemed the following aspects as especially important: (1) data sovereignty, (2) patients‘ protection-worthy interests, (3) patients‘ rights, and (4) compliance with ethical requirements for the protection of sensitive health data [14].

Both patients’ interests and their rights concerning the use of their routine medical data have been studied before. In a systematic review, Aitken et al [15] synthesized the results of qualitative research on data sharing for the purpose of health research. They identified overall widespread conditional support for this purpose. The conditions for support included, inter alia, the assurance of individuals’ confidentiality, a preference for the anonymity of data, and assurances of data security [15]. However, it is unclear whether these results and conditions can be applied to AI—a fundamentally new general-purpose technology. A study by McCradden et al [16] aimed to generate insights into public perceptions on using health data for AI research. They concluded that the general views about AI in their studied sample of the general population were mostly negative. Still, the participants were able to describe the potential benefits for health research that could be gained through the use of AI. Important conditions for supporting the use of health data for AI research were consent, the transparency of AI use, and assurances of data privacy [16]. These findings were based on a sample from the general population in Canada. It is plausible to assume that these preferences and opinions could vary among different cultural contexts and between the general population and current patients. Hence, we decided to involve patients in the first part of our project, even though they will not be direct users or beneficiaries of our generic infrastructure. To facilitate later discussions on patient and public involvement in research, the Guidance for Reporting Involvement of Patients and the Public [17] framework will be used.

The availability of large quantities of structured, high-quality data is fundamental to the development of AI tools [4,18]. Hospitals and other health care facilities inherently store the large amounts of data needed for AI development, but access to these data can pose legal and ethical challenges [12,13]. Besides that, legal certainty for the (partly) automized testing of routine medical data against a defined gold standard is missing [19]. Therefore, databases for the development and testing of new AI algorithms are often assembled manually. This requires high efforts from hospitals and product developers and slows down development and evaluation processes. In turn, patients can only benefit from new innovations after significant delays.

These problems and questions are addressed by the Protected Artificial Intelligence Innovation Environment for Patient-Oriented Digital Health Solutions for Developing, Testing, and Evidence-Based Evaluation of Clinical Value (pAItient) project, which aims to establish the AI Innovation Environment as a proof-of-concept extension of the already existing Medical Data Integration Center [20] at the Heidelberg University Hospital. The project partners include the German Cancer Research Center, the German Research Center for Artificial Intelligence, and Mint Medical GmbH (Heidelberg, Germany).

We also propose that knowledge of the requirements and needs of stakeholders, such as patients, health care providers, and industry partners, regarding the planned, generic infrastructure is an important antecedent for high acceptance and usefulness. This infrastructure is a novel concept and thus warrants a close analysis of factors influencing stakeholder acceptance.

### Aims

This paper presents the protocol for our study, which aims to explore stakeholders’ requirements for developing AI in partnership with an academic hospital and granting AI experts access to anonymized personal health data.

## Methods

We designed a multistep mixed methods study, combining qualitative and quantitative measures. This approach will allow us to gain in-depth insights into stakeholders’ opinions and quantify our findings.

### Stakeholders and Participants

Study participants were recruited from the following six stakeholder groups: (1) researchers from a biomedical research institute, (2) researchers from an AI research institute, (3) employees from start-up companies in the field of AI development, (4) employees from an AI imaging company, (5) patients at the Heidelberg University Hospital, and (6) physicians actively working in inpatient or outpatient health care.

### Participant Recruitment

Potential study participants from groups 1 to 4 were invited via email, following a snowball sampling approach. Patients (group 5) were recruited through a purposive sampling approach. Recruitment took place in different departments (the Department of Obstetrics and Gynecology, the Department of Internal Medicine, and the outpatient department at the National Center for Tumor Diseases Heidelberg) at the Heidelberg University Hospital, which is one of Europe’s largest medical centers [21]. Patients were approached by a study team member and invited to participate. In addition, informational materials, such as leaflets containing information on the study and the contact data of the study team, were provided. Recruitment procedures for physicians (group 6) are described elsewhere [22].

The inclusion criteria for groups 1 to 4 and group 6 were employment or the conduction of research activities in one of the defined areas. To be able to participate, patients had to report either a self-defined chronic disease or at least 3 visits to the hospital or an ambulatory health care provider in the last 3 months. The exclusion criteria were persons aged under 18 years and persons whose command of German or English was not sufficient for conducting an interview.

### Data Collection

**Figure 1:**
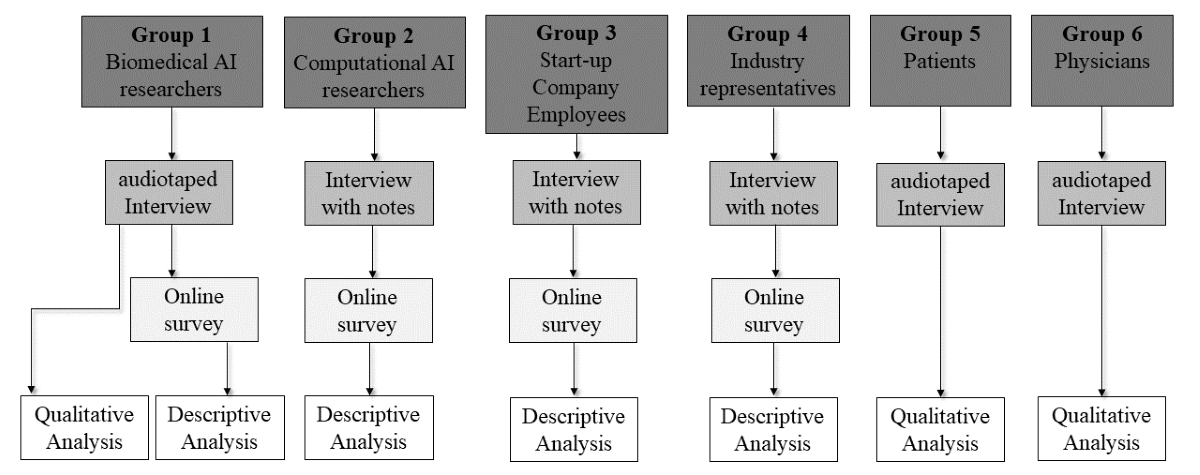
Study design overview. AI: artificial intelligence.

The following paragraphs describe the qualitative and quantitative methods that were used for data collection (Figure 1 depicts an overview). The methods used for data analysis are described in their respective paragraphs.

#### Qualitative Measures

Within an interprofessional team of researchers from the fields of health services research and medical informatics, semistructured interview guidelines were developed. The guidelines for professional interview partners were structured in 3 sections. The first section asked participants to briefly describe the projects they are currently working on. The second section contained questions concerning data usage, such as questions about the kinds of data that participants need for their projects. In the third section, participants were asked to elaborate on how an academic hospital could support them in their work. Translated versions of the interview guides can be found in Multimedia Appendix 1. A translated version of the interview guide for physicians was published elsewhere [22].

For patients, the interview guidelines followed a different approach. They included questions about general thoughts and expectations surrounding AI and the use of data for the development of AI. As these topics are complex and can be difficult to understand for patients, case vignettes were used for the patients’ interview guidelines.

Interviews with AI researchers, start-up company employees, and industry representatives were individually scheduled and were conducted by a researcher with a professional background in medical informatics. A researcher with profound experience in qualitative research methods was present during the interviews to facilitate data collection and to take notes.

Interviews with patients and physicians were conducted by researchers from the field of health services research. All interviews were performed via a videoconferencing tool. Participants were informed about the aims of the study before the interviews and were asked to give their informed consent. Interviews with participants from the biomedical research institute, patients, and physicians were audiotaped, pseudonymized, and transcribed verbatim, following appropriate transcription guidelines. These data were transcribed, managed, and analyzed with MAXQDA 2020 (VERBI Software GmbH). Participants from these groups were also asked to provide sociodemographic data, such as age and gender, on a separate form. After the data collection was completed, the data analysis was conducted according to thematic analysis procedures [23].

Interviews with participants from the remaining groups were documented through field notes. These were reviewed and used as the basis for developing the quantitative instrument.

#### Quantitative Measures

Web-based surveys were created based on the interview guidelines and field notes from the interviews. The questionnaires followed the same structure for all participant groups, but items varied and were tailored to each participant group. Within its base structure, the questionnaires included questions on the software, hardware, and types of data necessary for AI development. Additionally, participants were asked to rate experienced barriers and advantages of working with academic hospitals on Likert scales. REDCap (Research Electronic Data Capture; Vanderbilt University; hosted at the Heidelberg University Hospital) was used for study data collection and management. REDCap is a secure, web-based software platform that was designed to support data capture for research studies [24,25]. Potential participants were invited to fill in the survey via email. The survey results will be analyzed descriptively.

### Ethics Approval

The design for the stakeholder requirements analysis study was approved by the Ethics Committee of the Heidelberg University Hospital (approval number: S-241/2021) in March 2021.

## Results

The pAItient project received funding from the German Federal Ministry of Health in August 2020. We conducted 18 interviews, which enabled us to design a tailored survey instrument for participant groups 1 to 4. For the quantitative part of the study, 21 surveys were filled. Data collection concluded in summer 2022. Data analysis is planned to start in fall 2022. We plan to publish the results in winter 2022 to 2023.

## Discussion

The aim of our study is to explore stakeholders’ requirements for developing AI in partnership with an academic hospital and granting AI experts access to anonymized personal health data. We planned to include perspectives from a multitude of stakeholders, such as patients, physicians, AI researchers, and industry employees. We believe that following our 2-step mixed methods approach will allow us to identify the stakeholders’ priorities and will enable them to make important suggestions. We will be able to include these priorities and suggestions into the development process and into the technical infrastructure of the proposed AI Innovation Environment at our institution.

Data collection posed a significant challenge to our study. We faced difficulties in recruitment, especially for the group of patients. We believe that this may have been due to the complexity of the topic—AI—and a resulting low interest in research participation, which has been identified as a barrier before [26]. Hence, we redesigned the invitational leaflets, introduced a small financial compensation for participation, and had different members of the study team approach patients at different times of the day and in different departments. However, patient recruitment remained challenging. In order to comply with the agreed timelines for the overall project and to be able to start developing the AI Innovation Environment, participant recruitment had to be concluded in summer 2022.

## Appendix

Multimedia Appendix 1 Translated interview guide.

## Abbreviations

AI: artificial intelligence
pAItient: Protected Artificial Intelligence Innovation Environment for Patient-Oriented Digital Health Solutions for Developing, Testing, and Evidence-Based Evaluation of Clinical Value
REDCap: Research Electronic Data Capture

## Data Availability

The data sets that have been generated during the study are not publicly available due to privacy regulations but are available from the corresponding author on reasonable request.
