# Supplementary material for: Exploring Stakeholder Requirements to Enable the Research and Development of Artificial Intelligence Algorithms in a Hospital-Based Generic Infrastructure: Protocol for a Multistep Mixed Methods Study
Source: JMIR Res Protoc. 2022 Dec 16;11(12):e42208. doi: 10.2196/42208 (PMC9804098; doi:10.2196/42208)
Supplement: Multimedia Appendix 1 [file resprot_v11i12e42208_app1.pdf]

Appendix: Translated interview guides

Interview guide for interviewees from professional groups 1-4

Introduction:

Did you already hear about the planned development of an AI Innovation Environment at the Heidelberg University Hospital?

> If not: The "pAItient" project aims to establish an AI Innovation Environment at the Heidelberg University Hospital. Within this environment, it will be possible to develop and test new AI developments.

Today, I would like to talk to you about your previous experience with AI development and your expectations for the new AI Innovation Environment.

1. **Current projects**

- Could you please describe very briefly which projects dealing with the topics of AI and health care data you are currently working on (or planning)?
- Which hardware and software do you currently use for AI/ML?
- What are your or your organization's goals in these projects? (e.g., research, selling patents or licenses, etc…)

2. **Data usage**

- To what extent do you need data from the Heidelberg University Hospital or comparable institutions for your projects?
- What kind of data do you need?
- How could data from external sources support your projects?
- What are possible advantages of data from an academic hospital? In this context, how relevant is the data storage location, for example if data are stored in the hospital's or your infrastructure?
- What difficulties have you encountered in the past in the context of these projects?
- To what extent have you encountered barriers in working with these kind of data?
- Concerning intellectual property, what role does the data provider play?

3. **Data provider**

- What kind of support from the data provider would help you in conducting your projects?
- What are necessary requirements that have to be met in order for you to work within the infrastructure and framework conditions of the AI Innovation Environment?

Conclusion

Do you have any further recommendations for the AI Innovation Environment?

Are there any other thoughts you would like to share with us at this time?

Thank you so much for the conversation and your interesting opinions!

Interview guide for patients

Introduction:

Today, I would like to talk with you about the topic of "artificial intelligence" and data usage for the development of "artificial intelligence". What are your first associations with these topics?

Now, I would like to read you a short definition of artificial intelligence:

"Artificial intelligence", also called AI, is part of the subject area of informatics. AI means, that a computer is able to fulfill tasks under supervision and learn from data and observations, which would normally require human intelligence. They can adapt autonomously when being "fed" big amounts of data. On this basis and when confronted with specific questions, they are able to detect patterns and make predictions or decisions.

Through AI, a computer could perform the following tasks:

- suggest new songs based on known musical preferences
- process billings in a hospital administration in an automized manner
- compare moles with a big data base during skin cancer screening and suspect skin cancer

Do you have any questions regarding this explanation and the examples?

1. **Scenario: AI Innovation Environment**

Imagine you are a new patient at our hospital and came in for an appointment. Here you are informed about a new infrastructure for the development of AI technologies that is being tested at the hospital, in corporation with companies and other research institutions. Before your treatment, you will be asked if your data can be used for the development of AI.

What are your initial thoughts?

In general, which data would you release for the development of AI? Which data would you prefer not to release?

Would you like to be informed about the use of your data? Should your approval be collected beforehand?

What could lead to your refusal of releasing data? What could lead to your approval?

To what extent would these factors be different if the data were not supposed to be used to for AI development, but for „usual“ software?

Would your answers be different if external companies are involved? Or if only publicly funded research institutions are involved?

2. **Reflection**
   In the past 30 minutes, we talked about AI in medicine. What, if anything, have you learned from this conversation? Has something changed in your view on the topic of AI in medicine or data usage?

Conclusion

Do you have any further recommendations or thoughts on this topic?

Are there any other thoughts you would like to share with us at this time?

Thank you so much for the conversation and your interesting opinions!
